# Explainable Boosting Machine approach identifies risk factors for acute renal failure

**DOI:** 10.1186/s40635-024-00639-2

**Published:** 2024-06-14

**Authors:** Andreas Körner, Benjamin Sailer, Sibel Sari-Yavuz, Helene A. Haeberle, Valbona Mirakaj, Alice Bernard, Peter Rosenberger, Michael Koeppen

**Affiliations:** 1 Department of Anesthesiology and Intensive Care Medicine, University Hospital, Hoppe-Seyler-Straße 3, 72076 Tübingen, Germany; 2 Medical Data Integration Center, University Hospital Tübingen, Tübingen, Germany

## Abstract

**Background:**

Risk stratification and outcome prediction are crucial for intensive care resource planning. In addressing the large data sets of intensive care unit (ICU) patients, we employed the Explainable Boosting Machine (EBM), a novel machine learning model, to identify determinants of acute kidney injury (AKI) in these patients. AKI significantly impacts outcomes in the critically ill.

**Methods:**

An analysis of 3572 ICU patients was conducted. Variables such as average central venous pressure (CVP), mean arterial pressure (MAP), age, gender, and comorbidities were examined. This analysis combined traditional statistical methods with the EBM to gain a detailed understanding of AKI risk factors.

**Results:**

Our analysis revealed chronic kidney disease, heart failure, arrhythmias, liver disease, and anemia as significant comorbidities influencing AKI risk, with liver disease and anemia being particularly impactful. Surgical factors were also key; lower GI surgery heightened AKI risk, while neurosurgery was associated with a reduced risk. EBM identified four crucial variables affecting AKI prediction: anemia, liver disease, and average CVP increased AKI risk, whereas neurosurgery decreased it. Age was a progressive risk factor, with risk escalating after the age of 50 years. Hemodynamic instability, marked by a MAP below 65 mmHg, was strongly linked to AKI, showcasing a threshold effect at 60 mmHg. Intriguingly, average CVP was a significant predictor, with a critical threshold at 10.7 mmHg.

**Conclusion:**

Using an Explainable Boosting Machine enhance the precision in AKI risk factors in ICU patients, providing a more nuanced understanding of known AKI risks. This approach allows for refined predictive modeling of AKI, effectively overcoming the limitations of traditional statistical models.

## Background

Acute kidney injury (AKI) is a prevalent and critical organ dysfunction among ICU patients, with incidences reported between 16.7 and 57.9% in various studies [1, 2]. This condition not only prolongs ICU stays, but also significantly increases mortality rates, highlighting the need for a thorough understanding and management of AKI [3]. AKI signifies immediate physiological distress and predisposes patients to long-term complications, serving as a crucial indicator of overall health in critical care settings [4, 5]. In a comprehensive multinational study, approximately one in five patients develop AKI postoperatively after major surgery, indicating a significant health care burden [6]. Therefore, several different AKI prediction scores have been developed.

Recent advancements in machine learning (ML) have revolutionized the predictive modeling of AKI outcomes, offering a new paradigm in ICU management strategies. The integration of ML models and explainable algorithms, such as Explainable Boosting Machines (EBM), has the potential to enhance predictive accuracy. These models, including those based on ensemble methodologies and explainable frameworks [7–9], excel in analyzing complex, multidimensional data sets to unearth hidden patterns and risk factors in complex clinical conditions that lead to AKI. By leveraging a vast array of patient data, from basic demographics to intricate hemodynamic profiles, ML algorithms provide a depth of analysis unattainable with traditional statistical approaches [10]. The challenge, however, lies in demystifying the complexity of these models to ensure their findings are interpretable and actionable for clinicians, thereby bridging the gap between advanced data analytics and practical clinical application.

Our study employs an ML algorithm designed for interpretability to analyze a comprehensive cohort of ICU patients. We focus on a broad spectrum of variables, encompassing demographic information, hemodynamic parameters, and treatment-related factors, to investigate AKI risks. Utilizing an Explainable Boosting Machine [11, 12], our approach aims to identify nuanced interactions among risk factors. This method not only bolsters the predictive accuracy, but also elucidates the complex dynamics influencing AKI, offering clinicians a transparent and understandable model that can be directly applied to improve patient management and outcomes.

## Material and methods

### Study population and ethical considerations

Data for this study were systematically gathered from the medical records at the University Hospital of Tübingen. The Ethics Committee of the hospital (IRB# 512/2023BO1) sanctioned the study, granting an exemption from the need for informed consent due to the preservation of patient anonymity.

### Inclusion criteria and data extraction

Our study, spanning from 2018 to 2022, focused on patients from the Department of Anesthesiology and Intensive Care Medicine admitted to the ICU, excluding those who underwent cardiac surgery. Priority was given to patients with recorded central venous pressure (CVP) and mean arterial pressure (MAP) measurements. From an initial pool of 3,672 patients, 3,556 were selected for analysis, excluding 116 patients due to incomplete hemodynamic data. The selection process involved extracting relevant ICD-10 and OPS-codes based on the *German Disease Related Group* (DRG) system, emphasizing the identification of patients with acute kidney injury (AKI) based on ICD-10 codes (Code: N17). This facilitated the compilation of a detailed database comprising demographic data, ICU variables, and clinically verified AKI diagnoses. In the subgroup of patients with septic shock, we extracted the most likely source of primary infection based on the primary patient records in our patient data management system, categorizing the sources of infection into: respiratory, abdominal, urinary tract, bloodstream, soft tissue, central nervous system, device-related infections, or other.

### Data collection and hemodynamic variable assessment

Hemodynamic data were automatically recorded in the ICU's patient data management system. Based on SQL database query the CVP values were extracted. Then for each patient, the average MAP and CVP during their ICU stay were extracted and exported into a new database based on the case-ID number. The database containing the hemodynamic parameters was merged with ICD-10 and OPS-codes and exported to JMP for further analysis.

### Machine learning process using Explainable Boosting Machine

In this study, variables were consistently referred to as such for clarity, though they are often termed 'features' within ML literature. We trained an Explainable Boosting Machine (EBM), along with Gradient Boosting (GB) and Random Forest Classifier (RF). The dataset was split into 80% for training and 20% for testing purposes. Patients with missing data were excluded from the analysis. Boolean variables were converted to numerical values of zero and one.

A hyper-parameter optimization was conducted across all ML algorithms utilizing tenfold stratified cross-validation (CV) framework. The models' performance was gauged by the mean of balanced accuracy, the area under the receiver operating characteristic curve (ROC-AUC) and the F1-score for each CV fold. The models were optimized for the ROC-AUC score. The variable importance rankings and shape functions were consistent across both the training and test datasets, confirming the stability of our findings. Further validation was performed by training other ML models (GB and RF) and evaluating the variable importance rankings using SHAP. SHAP is a game theoretic approach to explain the output of ML models and finding the contributions of each variable to the model output.

The EBM's rationale is grounded in its construction from a generalized additive model (GAM) framework $g\left(y\right)= {\beta }_{0}+\sum {f}_{i}({x}_{i})$, where $g$ represents the link function and ${f}_{i}$ denotes the shape function for variable ${X}_{i}$. In classification contexts, the link function $f$ is typically a logistic function. The additive nature of the model allows each variable to contribute independently, facilitating straightforward interpretation of its influence on the prediction outcome. The incorporation of shape functions for each variable permits the modeling of complex, potentially non-linear associations with the predicted outcome. GAMs thus can achieve greater accuracy than simpler linear models. EBMs further integrate advanced ML techniques like bagging and boosting, yielding performance on par with leading ML methods such as GB and RF.

ML was performed in Python 3.8.10, using pandas 1.1.4, sklearn 1.2.0 (RF, GB, CV) [13] and interpret 0.3.0 (EBM) [14]. Variable importance rankings were evaluated using both interpret and shap 0.41.0 [15]. Visualization was done in RStudio 1.3.1093 using R 4.3.1, tidyverse 1.3.1 [16], ggpubr 0.6.0 [17], and patchwork 1.1.1 [18].

### Statistical analysis, and model assessment

The primary outcome was the diagnosis of acute kidney injury (AKI) during the ICU stay. Continuous variables were tested for normal distribution using the Shapiro‒Wilk goodness-of-fit test. Variables are reported as the mean ± standard deviation or the median (interquartile range), as appropriate. To complement our ML approach, we conducted univariate and multivariate logistic regression analyses to identify risk factors for AKI. Variables with a *p* value of less than 0.1 in the univariate analysis were included in the multivariate analysis. The Hosmer‒Lemeshow test assessed the model's goodness-of-fit, and the area under the receiver operating characteristic (ROC) curve was calculated. For normally distributed variables, Student's *t*-test was employed, while the Mann‒Whitney *U* test was used for non-parametric comparisons. The Chi-square independence test or Fisher's exact test was used for categorical variables. *P* values less than 0.05 were considered statistically significant. All statistical analyses were performed using Python for the ML components and JMP 16 (SAS Institute Inc., Cary, USA), Prism 9 (GraphPad Software Inc.) for other statistical evaluations.

## Results

### Analysis of demographic characteristics

Our comprehensive study analyzed demographic characteristics, clinical parameters, and risk factors in a cohort of 3572 critically ill patients to elucidate the determinants of acute kidney injury (AKI). Within this cohort, 848 patients developed AKI while 2724 did not. We observed a statistically significant higher mean age in the AKI group (64 ± 15 years) compared to those without AKI (59 ± 17 years, *p* < 0.0001). A gender disparity was also noted, with males presenting a greater likelihood of AKI, as supported by a lower proportion of females in the AKI group (8% vs. 35%, *p* < 0.0001). Additionally, renal replacement therapy (RRT) was required in 45.05% of patients with AKI compared to 2.24% of those without AKI (*p* < 0.0001), highlighting the severity of renal impairment in the AKI population (Table 1).

**Table 1:** Correlation between average central venous pressure and acute kidney injury in critically ill patients

	Total cohort (*n* = 3572)	With AKI (*n* = 848)	Without AKI (*n* = 2724)	*P*-value
*Demographics*				
Age (years; mean ± SD)	60 ± 16	64 ± 15	59 ± 17	** < 0.0001**
Female (no.; %)	1547 (43)	284 (8)	1263 (35)	** < 0.0001**
*Clinical parameters*				
Average CVP (mmHg; median [IQR])	9 (6–13)	12 (8–15)	8 (5–12)	** < 0.0001**
Average MAP (mmHg; median [IQR])	81 (77–85)	80 (76–85)	81(77–85)	0.1119
ICU length of stay (days; median [IQR])	14 (8–24)	23 (7–12)	12 (7–20)	** < 0.0001**
Maximal SOFA score (median [IQR])	6 (3–9)	9 (7–12)	5 (3–7)	** < 0.0001**
*Treatment variables*				
Blood transfusions (no.; %)	1796 (49)	689 (81)	1076 (40)	** < 0.0001**
ECLS (no.; %)	3 (0)	2 (.2)	1 (.4)	0.1422
*Organ dysfunction/shock*				
Cardiogenic shock (no.; %)	72 (2)	46 (5)	26 (1)	** < 0.0001**
Hypovolemic shock (no.; %)	188 (5)	108 (13)	80 (3)	** < 0.0001**
Septic shock (no.; %)	311 (9)	234 (27)	77 (3)	** < 0.0001**
Liver failure (no.; %)	184 (7)	219 (26)	41 (1.6)	** < 0.0001**
RV failure (no.; %)	64 (2)	37 (4)	27 (1)	** < 0.0001**
LV failure (no.; %)	184 (5)	89 (10)	95 (4)	** < 0.0001**
Coagulopathy (no.; %)	125 (3)	93 (11)	32 (1)	** < 0.0001**
Neurologic dysfunction (no.; %)	510 (14)	223 (27)	287 (11)	** < 0.0001**
Surgical subspecialties*				
Patients with upper GI Surgery (no.; %)	442 (12)	138 (16)	311 (11)	** < 0.0001**
Patients with Lower GI Surgery (no.; %)	547 (15)	137 (16)	305 (12)	**0.0001**
Patients with liver/biliary/pancreatic surgery (no.; %)	956 (26)	313 (37)	643 (24)	** < 0.0001**
Patients with intracranial surgery (no.; %)	1259 (35)	65 (8)	1194 (44)	** < 0.0001**
Patients with spinal surgery (no.; %)	186 (5)	43 (5)	143 (5)	0.8373
Patients with thoracic surgery (no.; %)	221 (6)	70 (8)	151 (6)	**0.0054**^**#**^
Patients with kidney/ureter surgery (no.; %)	239 (7)	69 (8)	170 (6)	0.0588^**#**^
Patients with vascular surgery (no.; %)	565 (16)	230 (27)	344 (12)	** < 0.0001**
Patients other surgeries (no.; %)	273 (8)	198 (12)	175 (7)	** < 0.0001**
*Comorbidities*				
Hypertension (no.; %)	1675 (47)	456 (54)	1219 (45)	** < 0.0001**
Diabetes mellitus (no.; %)	656 (18)	211 (25)	455 (16)	** < 0.0001**
Chronic kidney disease (no.; %)	280 (8)	133 (16)	147 (5)	** < 0.0001**
Ischemic heart diseases (no.; %)	419 (12)	147 (17)	272 (10)	** < 0.0001**
Neoplasms (no.; %)	1082 (30)	261 (31)	821 (30)	0.6412
*Outcomes*				
Stage 1 AKI (no.; %)		228 (27)		
Stage 2 AKI (no.; %)		217 (26)		
Stage 3 AKI (no.; %)		444 (52)		
*Renal replacement therapy*				
Yes (no.; %)	443 (12)	382 (45)	61 (2)	** < 0.0001**^**#**^
No (no.; %)	3129 (88)	466(55)	2663 (98)	

*CVP*  central venous pressure, *AKI*  acute renal failure, *SD*  standard deviation, *IQR*  interquartile range, *SOFA*  Sequential Organ Failure Assessment, *ICU*  Intensive Care Unit. The cohort is divided based on the presence of acute renal failure as determined by ICD codes. *P*-values indicate the level of statistical significance for differences between patients with and without AKI

^*^Patients may be represented in more than one surgical category if they underwent multiple procedures

^#^Compared by Fisher’s exact test

### Analysis of infection sources in septic shock

Among 311 patients with septic shock, abdominal infections were most common (57.6%), followed by respiratory (17.7%) and urinary tract infections (5.8%). In patients with AKI, 58.6% had abdominal infections compared to 54.6% without AKI (*p* = 0.5954). Respiratory infections were observed in 16.7% with AKI versus 20.8% without AKI (*p* = 0.3957). Other infection sources, including bloodstream, soft tissue, CNS, and device-related infections, showed no significant differences between groups (Table 2).

**Table 2:** Subgroup with septic shock—sources of infection

Source of infection	Total (*n* = 311)	With AKI (*n* = 234)	Without AKI (*n* = 77)	*P*-value
Respiratory infections no (%)	55 (17.7)	39 (16.7)	16 (20.8)	0.3957^#^
Abdominal infections no (%)	179 (57.6)	137 (58.6)	42 (54.6)	0.5954^#^
Urinary tract infections no (%)	18 (5.8)	11 (4.7)	7 (9.1)	0.1641^#^
Bloodstream infections no (%)	14 (4.5)	11 (4.7)	3 (3.9)	1.0000^#^
Soft tissue infections no (%)	15 (4.8)	11 (4.7)	4 (5.2)	0.7688^#^
Central nervous system infections no (%)	5 (1.6)	3 (1.3)	2 (2.6)	0.6006^#^
Device-related infections no (%)	6 (1.9)	6 (2.6)	0 (0)	0.3422^#^
Other no (%)	19 (6.1)	15 (6.4)	4 (5.2)	0.5780^#^

^#^Compared by Fisher’s exact test

### Risk factor assessment

Our multivariate logistic regression analysis highlighted central venous pressure (CVP) as a pivotal factor, with a marked increase in the odds of AKI corresponding to each mmHg rise in average CVP (adjusted OR = 1.07, 95% CI: 1.05, 1.08, *p* < 0.0001). Age increment per year was associated with a slight but significant increase in AKI risk (adjusted OR = 1.01, 95% CI: 1.00, 1.02, *p* = 0.00512) (Table 3). Males had a higher risk compared to females (adjusted OR = 1.31, 95% CI: 1.07, 1.60, *p* = 0.0086), aligning with the demographic distribution (Table 1).

**Table 3:** Univariate and multivariate logistic regression analysis of factors associated with acute kidney injury

Variable	Univariate OR (95% CI)	*P*-value	Adjusted OR (95% CI)	*P*-value
Average CVP [mmHg]	1.11 (1.09, 1.13)	** < 0.0001**	1.07 (1.05, 1.08)	** < 0.0001**
Average MAP [mmHg]	0.99 (0.98, 1.00)	**0.0131**	1.00 (0.99, 1.01)	0.68831
Age [years]	1.02 (1.02, 1.03)	** < 0.0001**	1.01 (1.00, 1.02)	**0.00512**
**Gender**				
Female	1 (Reference)	–	1 (Reference)	–
Male	1.72 (1.46, 2.01)	** < 0.0001**	1.31 (1.07, 1.60)	**0.0086**
**Comorbidities**				
Hypertension	1.44 (1.23–1.68)	** < 0.0001**	1.26 (1.10, 2.02)	**0.0103**
Diabetes mellitus	1.69 (1.40, 2.04)	** < 0.0001**	1.25 (0.98,1.59)	0.7558
Chronic kidney disease	3.26 (2.54, 4.18)	** < 0.0001**	1.49 (1.10, 2.02)	** < 0.0001**
Heart failure	3.53 (2.67, 4.67)	** < 0.0001**	1.65 (1.15, 2.38)	**0.0072**
Ischemic heart disease	3.26 (2.54, 4.18)	** < 0.0001**	0.75 (1.57, 0.99)	**0.0463**
Arrhythmias	3.02 (2.53, 3.60)	** < 0.0001**	1.60 (1.27, 2.01)	** < 0.0001**
Cerebrovascular disease	2.24 (1.72, 2.92)	** < 0.0001**	1.30 (0.89, 1.89)	0.1679
COPD	1.81 (1.24, 2.64)	** < 0.0001**	1.04 (0.66, 1.62)	0.8738
Asthma	1.00 (0.49, 2.05)	0.9915		
Pulmonary hypertension	3.03 (1.75, 5.25)	** < 0.0001**	1.06 (0.53, 2.08)	0.8720
Thyroid disorders	1.34 (1.11, 1.63)	**0.0031**	1.04 (0.82, 1.32)	0.7232
Liver disease	6.45 (5.33, 7.80)	** < 0.0001**	4.50 (3.54, 5.73)	** < 0.0001**
Anemia	8.06 (6.62, 9.82)	** < 0.0001**	3.80 (3.03, 4.74)	** < 0.0001**
Dementia	1.98 (0.93, 4.20)	0.0873		
Parkinson’s disease	1.88 (0.74, 4.79)	0.2006		
Rheumatoid arthritis	0.60 (0.17, 2.06)	0.3928		
Neoplasm				
*Surgical subspecialties*				
Upper GI surgery	1.53 (1.22, 1.90)	**0.0002**	1.23 (0.94, 1.60)	0.1251
Lower GI surgery	2.94 (2.42, 3.55)	** < 0.0001**	1.72 (1.36, 2.16)	** < 0.0001**
Liver/biliary/pancreatic surgery	1.89 (1.61, 2.23)	** < 0.0001**	0.90 (0.71, 1.14)	0.3701
Intracranial surgery	0.11 (0.08, 0.14)	** < 0.0001**	0.35 (0.24, 0.51)	** < 0.0001**
Spinal surgery	0.96 (0.68, 1.37)	0.8373	0.99 (0.64, 1.53)	0.9767
Thoracic surgery	1.53 (1.14, 2.05)	0.3928		
Kidney/ureter surgery	1.33 (0.99, 1.78)	0.0588		
Vascular surgery	2.60 (2.15, 3.15)	** < 0.0001**	1.52 (1.20, 1.92)	**0.0005**

**Fig. 1:**
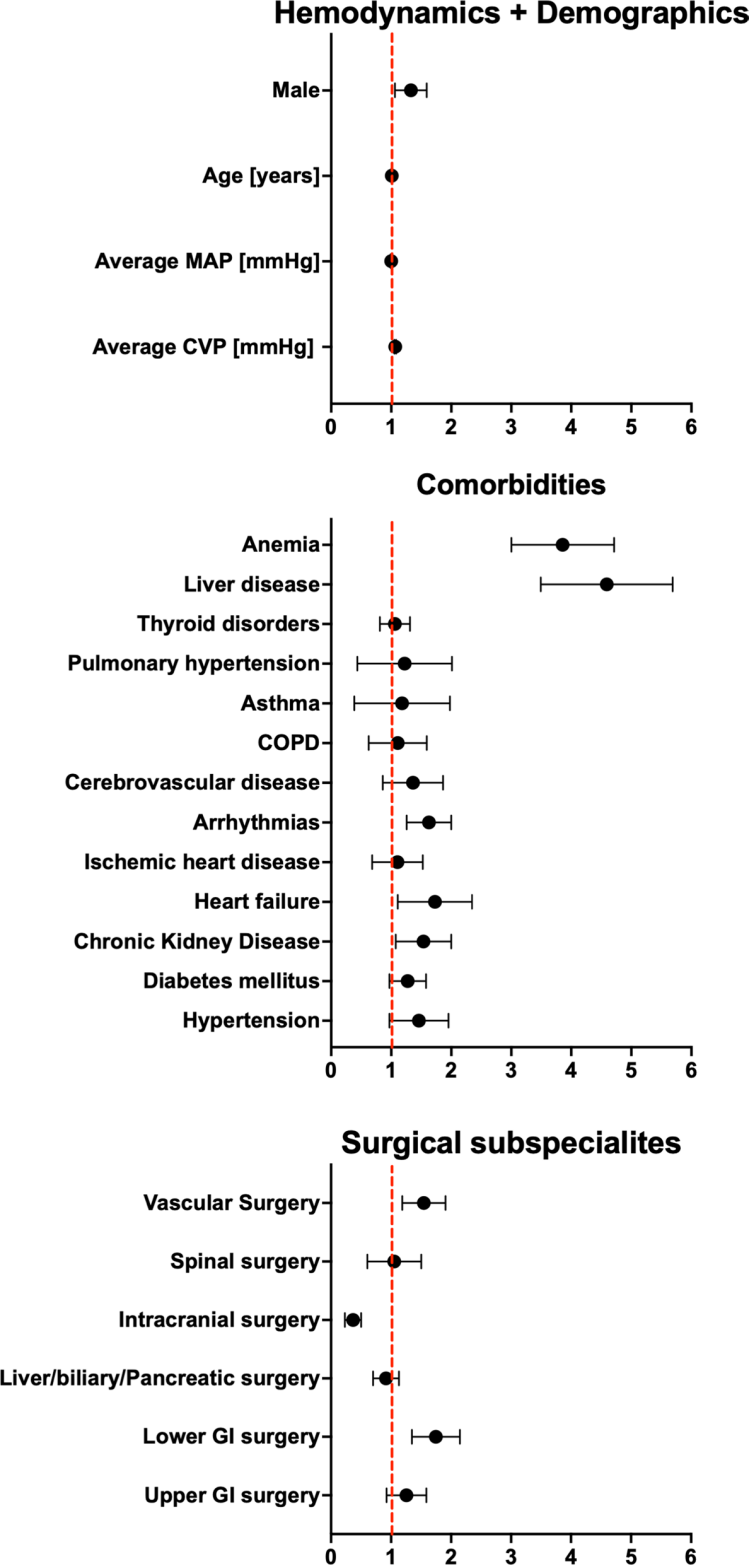
Forrest plot depicting odds ratios (OR) and 95% Wald confidence intervals (CI) derived from multivariate logistic regression analysis (Table 3). The OR estimates illustrate the association between various covariates and the likelihood of acute kidney injury in critically ill patients. Each point estimate on the plot corresponds to an individual covariate, while the horizontal lines represent the 95% CI. Notable findings include a significant association between average CVP, age, male gender, comorbidities (hypertension, chronic kidney disease, heart failure, ischemic heart disease, arrhythmias, pulmonary hypertension, thyroid disorders, liver disease, anemia), and certain surgical subspecialties (lower GI surgery, intracranial surgery, spinal surgery, vascular surgery) with acute kidney injury risk

Comorbidities such as hypertension, chronic kidney disease, heart failure, and arrhythmias were identified as significant risk factors for AKI (Table 3). The impact of surgical subspecialties revealed that lower gastrointestinal (GI) surgery and vascular surgery were notably linked with an increased risk of AKI (adjusted OR = 1.72 and 1.52, respectively, *p* < 0.0001 for both) (Table 3). Conversely, liver/biliary/pancreatic surgery and intracranial surgery were not associated with a significant adjusted risk (Fig. 1).

### Risk factor assessment

Our multivariate logistic regression analysis highlighted central venous pressure (CVP) as a pivotal factor, with a marked increase in the odds of AKI corresponding to each mmHg rise in average CVP (adjusted OR = 1.07, 95% CI: 1.05, 1.08, *p* < 0.0001). Age increment per year was associated with a slight but significant increase in AKI risk (adjusted OR = 1.01, 95% CI: 1.00, 1.02, *p* = 0.00512) (Table 3). Males had a higher risk compared to females (adjusted OR = 1.31, 95% CI: 1.07, 1.60, *p* = 0.0086), aligning with the demographic distribution (Table 1).

Comorbidities such as hypertension, chronic kidney disease, heart failure, and arrhythmias were identified as significant risk factors for AKI (Table 3). The impact of surgical subspecialties revealed that lower gastrointestinal (GI) surgery and vascular surgery were notably linked with an increased risk of AKI (adjusted OR = 1.72 and 1.52, respectively, *p* < 0.0001 for both) (Table 3). Conversely, liver/biliary/pancreatic surgery and intracranial surgery were not associated with a significant adjusted risk (Fig. 1).

### Explaining acute renal failure risk with advanced machine learning

**Fig. 2:**
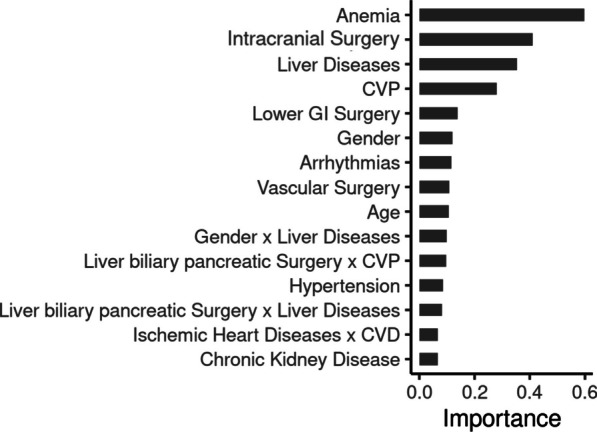
Variable importance in predicting AKI as determined by Explainable Boosting Machine. This figure illustrates the weighted mean absolute scores of various clinical and demographic factors in predicting the development of acute kidney injury (AKI) using an Explainable Boosting Machine model. Each bar represents a variable's weighted contribution to the model, with longer bars indicating a greater importance. Factors include patient demographics, pre-existing conditions, and surgical history, such as anemia, liver diseases, and different types of surgeries (e.g., intracranial surgery). Combination of two parameters are linked by an ‘*x*’. The score reflects the strength of association with AKI risk after adjustment for covariates within the model

**Fig. 3:**
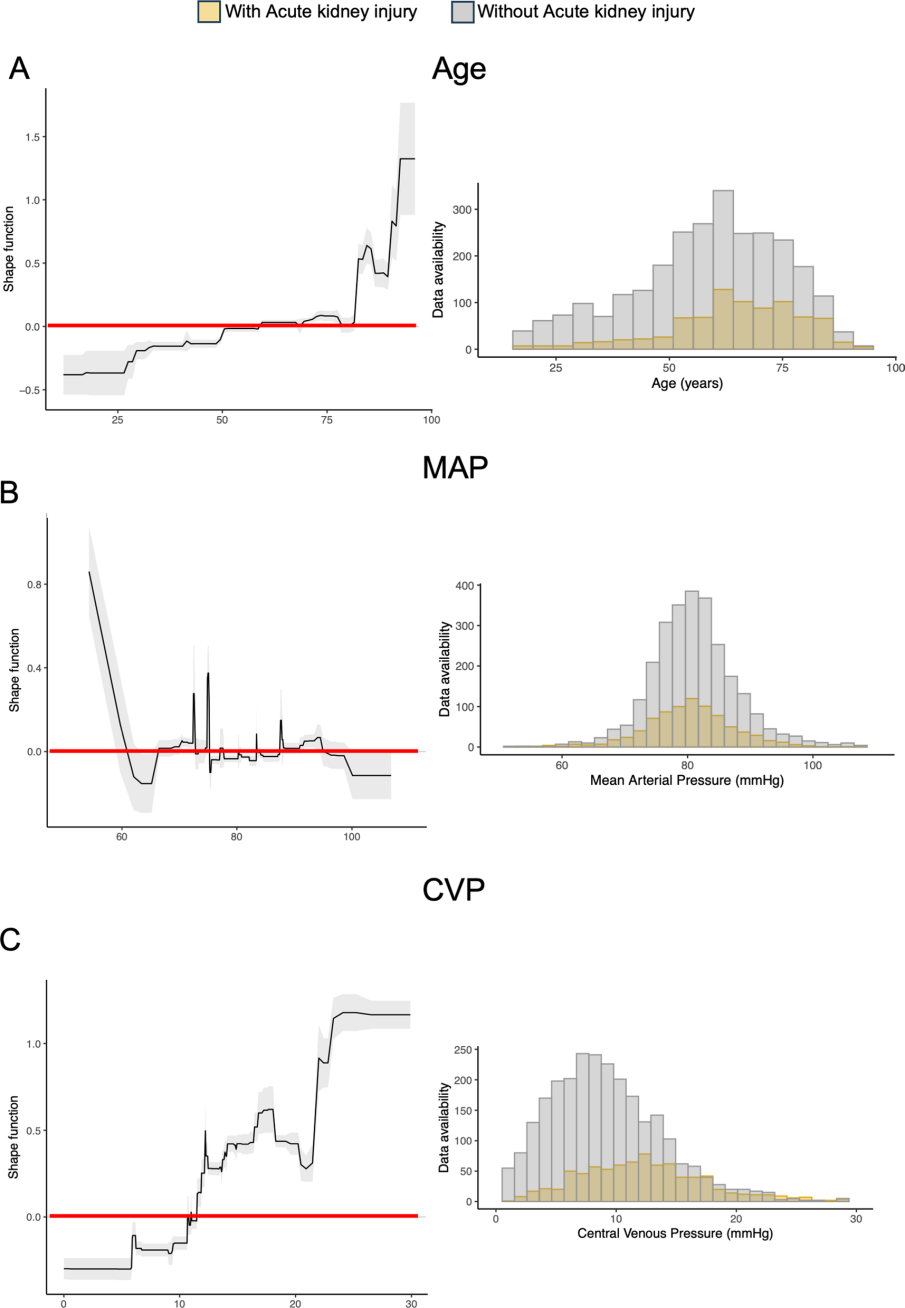
Impact of age, mean arterial pressure (MAP), and central venous pressure (CVP) on the development of acute kidney injury (AKI). Each step in the score lines represents a change in the predictive importance of the respective variable, with positive values indicating higher predictive importance for AKI development (0 is indicated by the red line in each shape function), histograms indicate the distribution of each variable within the study population. Analysis conducted using an Explainable Boosting Machine, with the score reflecting the strength of association with AKI risk after adjusting for covariates. **A** Panel displays the relationship between patient age and the score assigned by the Explainable Boosting Machine, indicating the relative importance of age in predicting AKI development. **B** The middle panel represents the association between the average mean arterial pressure and the predictive score, with the corresponding density distribution of MAP values. **C** Illustrates the correlation between average central venous pressure and the predictive score, alongside the density distribution of CVP measurements

To supplement conventional statistical methods, we employed an advanced ML model, which identified anemia, neurosurgical intervention, liver disease, and mean CVP as key variables influencing AKI risk (Fig. 2). Age emerged as a variable of interest, with the likelihood of AKI rising substantially after the age of 50 and sharply escalating beyond 80 years. In terms of hemodynamic parameters, a mean arterial pressure (MAP) below 60 mmHg was strongly associated with AKI, whereas a MAP above 65 mmHg was not, suggesting a threshold effect (Fig. 3B). A noteworthy CVP threshold was identified at 10.7 mmHg, beyond which the risk of AKI significantly increased (Fig. 3C). These findings were in line with our regression analysis, emphasizing the significance of elevated CVP in the context of AKI risk.

Focusing on the performance metrics of the Explainable Boosting Machine (EBM), we observed a balanced accuracy score of 0.72 and a ROC-AUC score of 0.88 (confidence interval: 0.86 to 0.89). Additionally, the EBM exhibited a specificity of 0.95, indicating its capability to accurately identify patients without AKI. This level of precision supports the insights generated by the model and aligns well with our regression analysis findings.

## Discussion

In this study, we investigated the determinants of acute kidney injury (AKI) in a diverse cohort of 3572 critically ill patients. Our initial statistical analysis highlighted age and gender as significant factors influencing AKI onset. Furthermore, we observed a strong link between hemodynamic parameters, particularly mean central venous pressure (CVP), and increased AKI risk. Leveraging insights from our initial analysis, we employed an Explainable Boosting Machine (EBM), a sophisticated ML model, to delve deeper into the associations and predictive variables related to AKI risk. This approach provided additional insights into the dynamics involved in AKI development among critically ill patients.

The impact of hemodynamic changes on kidney vulnerability is well-established in the literature. Prior studies have demonstrated a significant relationship between AKI and sustained low mean arterial pressures (MAP) [19, 20]. In alignment with these findings, our analysis showed that a MAP below 60 mmHg markedly increased the risk of AKI. Additionally, our study brought to light a less commonly explored correlation: the association between high CVP values and kidney injury. Specifically, we found that a CVP exceeding 10.7 mmHg was significantly associated with AKI, consistent with recent observations in cardiothoracic and critically ill patient populations [21–23]. The identification of a critical CVP threshold of 10.7 mmHg by our ML analysis further refined our understanding of the hemodynamic influences on AKI. Interestingly, our analysis indicated that an increase in mean arterial pressure (MAP) towards hypertensive values appears to be protective or at least not harmful in the context of AKI. This observation suggests that maintaining a higher MAP may mitigate the risk of AKI in critically ill patients.

Our analysis also identified neurosurgical interventions as being associated with a lower risk of AKI compared to other surgeries. This finding contrasts with some studies that suggest emergency neurosurgical procedures might carry a significant risk of AKI [24, 25]. The lower risk observed in our cohort could be due to several factors, including differences in patient management and surgical techniques. This finding highlights the need to consider risk factors within specific patient populations and clinical settings.

Our analysis utilized the SHAPE functions. They provide a visual representation of the influence of continuous variables on AKI risk. This tool allowed us to identify critical threshold values, such as the CVP, and offers practical insights for clinical decision-making. The SHAPE function's ability to illustrate variable impact in an interpretable manner is a useful tool in the analysis of continuous data in retrospective studies.

Our analysis also reaffirmed the role of gender and age as significant predictors of AKI, with males showing a higher risk compared to females, a finding supported by previous research [26, 27]. Moreover, an experimental study suggested that testosterone might increase susceptibility to ischemic renal injury [28]. These insights, along with our hemodynamic observations, align with existing literature, thereby independently confirming previous studies that identified these factors as major risks for AKI development [29, 30].

The application of ML algorithms for AKI prediction has evolved significantly over recent years. Various methods, including Extreme Gradient Boosting (XGBoost), Gradient Boosting Machine, Support Vector Machine (SVM), Decision Tree, and Artificial Neural Network, have been explored, all demonstrating robust AKI prediction capabilities, often surpassing traditional linear models [7, 31–33]. Unlike these studies that utilize publicly available ICU patient databases such as MIMIC-IV for training and testing, our study uses a proprietary cohort, making it a distinctive contribution by providing a real-world analysis. Furthermore, our study addresses the issue of ML complexity, which often limits interpretability for clinicians. To this end, we have adopted the Explainable Boosting Machine (EBM), which uniquely combines high predictive accuracy with transparent, interpretable insights into the model's decision-making processes [34]. This level of clarity is especially valuable in clinical settings, where understanding the rationale behind predictions is as crucial as the predictions themselves. Consequently, our model not only predicts AKI with high accuracy, but also enables a detailed, step-wise analysis as depicted in our Fig. 3.

Despite these novel insights, our study has limitations. We did not fully explore the mechanisms linking high CVP to kidney injury, although prevailing theories suggest that venous congestion may impair renal blood flow by reducing arterial–venous pressure gradients, potentially leading to congestive renal injury [21]. This hypothesis is supported by the observed correlation between conditions characterized by impaired venous drainage, such as liver disease, and AKI in our study [35, 36]. Moreover, our reliance on retrospective data to train our ML model introduces the inherent limitations of such studies, including the inability to establish causation. Another limitation of our study is the granularity of comorbidities captured in our dataset. Due to the retrospective nature of our data collection, the level of detail for comorbid conditions is restricted to billing codes within the German Diseases Related Group system. This limitation prevented us from analyzing different subgroups of comorbidities, such as various grades of hypertension, which could provide more nuanced insights into AKI risk factors. Future clinical trials are needed to explore whether the variables identified as potential therapeutic targets, like elevated CVP, can effectively reduce AKI risk.

In conclusion, our comprehensive investigation enriches the understanding of AKI in critically ill patients by integrating conventional risk factor analysis with advanced ML techniques. By identifying key determinants of AKI and employing the EBM for in-depth analysis, our study highlights the importance of a multifaceted approach to AKI risk assessment. Interpretable ML models have the potential to improve clinical decision-making in ICU settings, contributing to targeted and effective AKI management strategies aimed at enhancing patient outcomes in critical care.

## Data Availability

The datasets used and/or analyzed during the current study are available from the corresponding author on reasonable request.
